# 

**Published:** 1871-12

**Authors:** 


### The Chicago Medical Examiner

A MONTHLY JOURNAL,

DEVOTED TO THE

Educational, Scientific, and Practical Interests

OF

### THE MEDICAL PROFESSION.

EDITED BY

N. S. DAVIS, M. D., and F. H. DAVIS, M. D.

---

### *PROSPECTUS FOR 1872.*

With the number for January, 1872, THE EXAMINER will enter upon its *thirteenth volume.* The publishers hope, by strict attention to the interests and requirements of their subscribers, to still further merit the very liberal and constantly increasing patronage and support which the profession throughout the country have extended to them during the past twelve years

### ITS CONTENTS

Will be of such a character as will directly aid the Physician in the daily practical duties of his profession. A large list of contributors, including many of the most eminent Physicians and Surgeons in the West, insures to THE EXAMINER an abundant supply of *Original Articles.* Each number will contain, in addition, *from 12 to 14 pages of Clinical Reports,* including abstracts of all the principal Medical and Surgical Cliniques of the Mercy Hospital, the Cook County Hospital, and the Free Dispensaries.

### The Select Department

Will contain a careful digest of the Professional and Scientific News and Literature, both at home and abroad.

---

**TERMS: $3.00 Per Annum, Payable Invariably in Advance.**

No name will be entered on the Books until the money has been received.

---

☞ *Liberal Commissions* offered to those who will interest themselves in obtaining new subscribers for THE EXAMINER. For every Club of four new subscribers, and $12, we will send an extra copy of THE EXAMINER, gratis, for one year.

---

SPECIAL NOTICE TO NEW SUBSCRIBERS.

To all new subscribers who will send their subscription for 1872 to this of[illegible] before December, 1871, we will mail the Oct., Nov. and Dec. numbers free.

[illegible]UBLISHERS CHICAGO MEDICAL EXAMINER,

797 Wabash Ave., Chicago, Ill.

